# Spatially precise chemo-radio-immunotherapy by antibody drug conjugate directed tumor radiosensitization to potentiate immunotherapies

**DOI:** 10.1038/s41698-025-00885-x

**Published:** 2025-04-04

**Authors:** Mahsa Mortaja, Stephen R. Adams, Rana R. McKay, J. Silvio Gutkind, Sunil J. Advani

**Affiliations:** 1 Department of Radiation Medicine and Applied Sciences, University of California San Diego, La Jolla, CA 92093 USA; 2 Department of Pharmacology, University of California San Diego, La Jolla, CA 92093 USA; 3 Department of Medicine, University of California San Diego, La Jolla, CA 92093 USA; 4 UC San Diego, Moores Cancer Center, La Jolla, CA 92093 USA; 5 Department of Urology, University of California San Diego, La Jolla, CA 92093 USA

**Keywords:** Radiotherapy, Targeted therapies, Radiotherapy, Targeted therapies

## Abstract

Concurrent chemo-radiotherapy is standard of care for locally advanced cancer patients. While radiotherapy and immuno-oncology have advanced precision oncology, chemotherapies in the chemo-radiotherapy paradigm remain non-targeted cytotoxins. Antibody drug conjugates offer an opportunity for targeted radiosensitization that stimulates immune responses while protecting normal tissues. Here, we discuss the rationale for combining antibody drug conjugates, radiotherapy and immunotherapies and opportunities for clinical translation to advance towards targeted chemo-radio-immunotherapy precision cancer care.

## The concurrent chemo-radiotherapy paradigm

While early stage cancers are effectively cured with surgery and radiotherapy, locally advanced cancers pose therapeutic challenges. The infiltrative nature of such aggressive cancers into adjacent normal tissues and/or lymph nodes precludes complete surgical resection or ablative radiotherapy techniques. Though potentially curable, these cancers remain a major driver of cancer mortality and treatment morbidity. To comprehensively tackle the known macroscopic tumor, regional lymph node spread and potential metastatic seeding, the concurrent chemo-radiotherapy paradigm was developed over forty years ago^1^. Rationales for combining focal ionizing radiation (IR) with systemically delivered drugs include: 1) Certain chemotherapies function as radiosensitizers by increasing IR induced DNA damage. 2) Independent mechanisms of tumor kill by chemotherapy and radiotherapy increase the probability of tumor eradication. 3) Spatial cooperativity with radiotherapy directed at visualized tumors and chemotherapy attacking microscopic spread out of the irradiated field. 4) Dose dense therapy with simultaneous administration of IR and chemotherapy maximizing upfront tumor kill thereby reducing the emergence of resistant tumor clonogens. Such multimodal cooperative tumor killing optimizes the therapeutic index by allowing for the use of lower more tolerable synergizing doses of chemotherapy and radiotherapy.

Through the 1980-1990’s, paradigm defining randomized controlled trials (RCTs) consistently demonstrated improved tumor control and/or patient survival when chemotherapy was given with radiotherapy (Table 1)^2–9^. The strength of concurrent chemo-radiotherapy is evident by superior patient outcomes across diverse tumor histologies including brain, head and neck (HNC), esophagus, lung, bladder and cervical cancers. Moreover for patients with HNC, bladder, rectal and anal cancers, chemo-radiotherapy protocols offer organ sparing approaches as an alternative to large en bloc resections that negatively impact survivorship quality of life^7,8,10,11^. While the IR in these seminal RCTs was delivered based on bony anatomy, radiotherapy planning has grown increasingly sophisticated^12^. Intensity modulated radiotherapy (IMRT) conforms IR deposition to tumor volumes identified by positron emission tomography-computed tomography (PET-CT) and magnetic resonance imaging (MRI). Image guided radiotherapy (IGRT) ensures each daily IR fraction is delivered as planned over the course of multiple weeks thereby minimizing peri-tumoral normal tissue damage. Advancing radiotherapy solutions further, proton beam radiotherapy with its Bragg peak increases the spatial conformality of IR damage to tumors^12^.

**Table 1:** Improved tumor control with concurrent chemo-radiotherapy

Cancer	Chemotherapy given with RT	Patient outcomes	Targetable receptor	FDA approved ADC
GBM	Temozolomide	2 yr OS^2^2: RT 10.9% *CRT 27.2%*		
Head and Neck	Cisplatin, carboplatin, 5-FU	3 yr OS^3^: RT 31% *CRT 51%*	TF	TV
Esophageal	Cisplatin, carboplatin, 5-FU, paclitaxel	5 yr OS^4^: RT 0% *CRT 26%*	HER2	T-DXd, T-DM1
NSCLC	Cisplatin, carboplatin, vinblastine, paclitaxel, etoposide	5 yr OS^5^: sequential CRT 10% *concurrent CRT 16%*	HER2 TF	T-DXd, T-DM1, TV
Rectal	5-FU, capecitabine	5 yr LF^6^: RT 16.5% *CRT 8.1%*	HER2 TF	T-DXd, T-DM1, TV
Anal	MMC, 5-FU	LF^7^: RT 59% *CRT 36%*		
Bladder	Cisplatin, MMC, 5-FU	2 yr DFS^8^: RT 54% *CRT 67%*	Nectin-4 Trop-2	EV, SG
Cervical	Cisplatin, 5-FU	5 yr OS^9^: RT 58% *CRT 73%*	TF	TV

*5-FU* 5-Fluorouracil, *LF* Local failure, *T-DM1* Trastuzumab emtansine, *ADC* Antibody drug conjugate, *MMC* Mitomycin C, *T-DXd* Trastuzumab deruxtecan, *CRT* Chemo-radiotherapy, *NSCLC* Non-small cell lung cancer, *TF* Tissue factor, *DFS* Disease free survival, *OS* Overall survival, *Trop-2* Trophoblast antigen 2, *EV* Enfortumab vedotin, *RT* Radiotherapy, *TV* Tisotumab vedotin, *GBM* Glioblastoma, *SG* Sacituzumab govitecan.

**Fig. 1:**
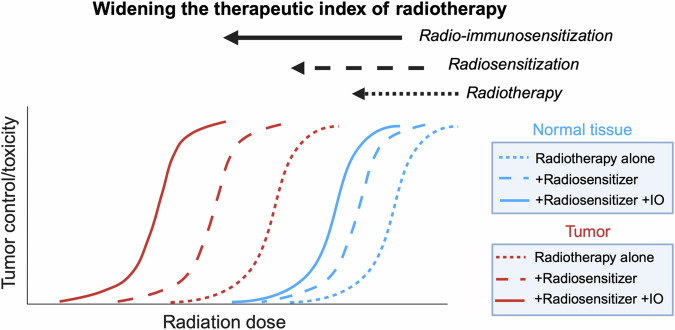
Increasing the therapeutic index of radiotherapy. The intrinsic therapeutic index of radiotherapy for cancer can be improved with tumor targeted radiosensitizers that preferentially sensitize cancer cells to radiotherapy. Radiosensitized tumor kill can stimulate anti-tumor immune responses and potentiate immunotherapies to further widen the therapeutic index.

Despite the progress in radiotherapy delivery and molecular targeted therapies, drugs in continued use with radiotherapy remain non-targeted cytotoxins from the advent of the chemo-radiotherapy era and include anti-metabolites (5-fluorouracil, gemcitabine), alkylating agents (cisplatin, carboplatin, temozolomide), anti-mitotics (vincristine, vinblastine, docetaxel, paclitaxel), topoisomerase inhibitors (etoposide) and anti-tumor antibiotics (mitomycin C)^13^. Although such chemotherapies improve tumor control (Table 1), non-targeted cytotoxins increase normal tissue damage in the irradiated field and have systemic toxicities. Therapy-induced side effects cause treatment delays and dose reductions that negatively impact tumor control. Additionally, non-targeted chemotherapies do not leverage vulnerabilities within tumor cells or specifically target alterations that drive tumor pathogenesis. Finally, dose-limiting toxicities preclude treatment intensification to further improve tumor control. Moving away from non-targeted chemotherapies towards tumor-directed radiosensitizers may widen the therapeutic index of chemo-radiotherapy and potentiate immunotherapies (Fig. 1).

## Tumor radiosensitization

The therapeutic index of radiotherapy is driven by DNA double strand break induction preferentially causing chromosomal aberrations and mitotic catastrophe in irradiated cancer cells over normal tissues (Fig. 1)^14^. With increasing IR dose there is a sigmoidal increase in both tumor control probability and normal tissue damage. Increased destruction of tumors over normal tissue defines radiotherapy’s therapeutic index based on differential DNA damage and subsequent repair capacity. Radiosensitizers increase tissue sensitivities to IR shifting the sigmoidal dose-response curve to the left (i.e. increased tumor kill and/or normal tissue damage at a given IR dose). To be clinically useful, radiosensitizers must sensitize tumors to IR to a greater extent than normal tissue, thereby widening the therapeutic index^14–16^. Mechanistic understanding of DNA damage responses has identified signaling pathways that can be pharmacologically inhibited resulting in increased IR kill^12,17^. However, clinical efficacy of pathway specific radiosensitizers has proven difficult as exemplified by epidermal growth factor receptor (EGFR) inhibition. EGFR activation promotes resistance to radiotherapy while inhibition with antibody (i.e. cetuximab) radiosensitizes cancer cells. However in RCTs, cetuximab combined with radiotherapy resulted in inferior tumor control compared to non-targeted cytotoxic cisplatin-radiotherapy^18^. Treatment intensification by adding cetuximab to standard chemo-radiotherapy regimens also failed to benefit patients^19,20^.

**Fig. 2:**
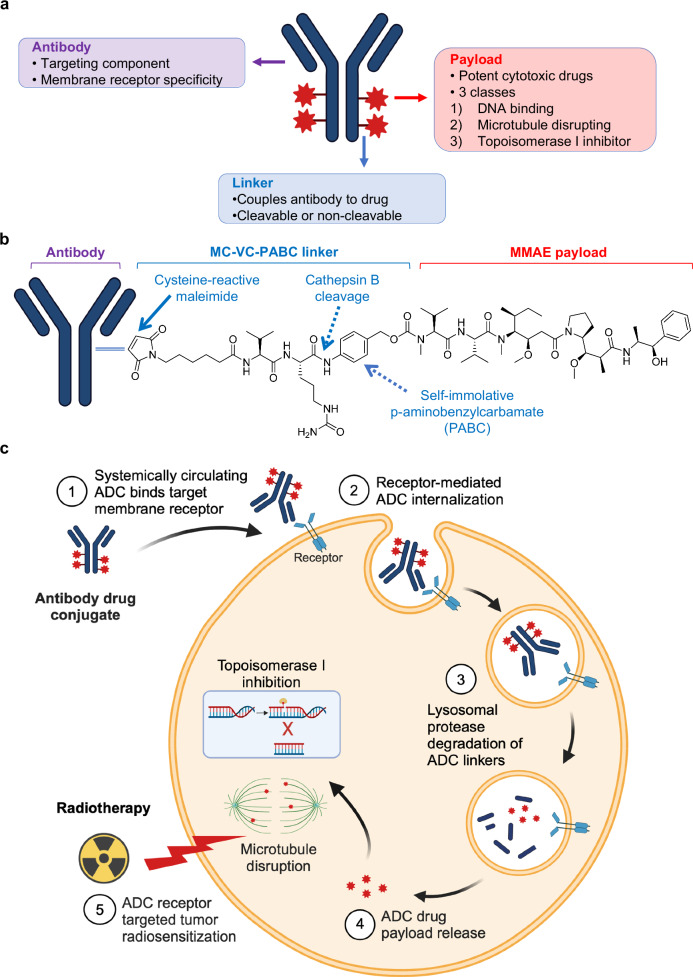
Antibody drug conjugates and radiosensitization. **a** Components of antibody drug conjugates. Targeting antibody has high affinity for receptors overexpressed on cancer cell membranes. Potent cytotoxic drug payloads attack fundamental cellular functions resulting in death. Linkers chemically attach targeting antibody to cytotoxic drugs. **b** Schema of MMAE ADCs with a cathepsin cleavable valine-citrulline dipeptide linker coupling MMAE to targeting antibody. **c** Sequential steps of ADC mediated cancer cell kill and radiosensitization. Systemically administered ADCs bind target membrane tumor cell receptors and undergo intracellular uptake through receptor mediated endocytosis. Linker degradation within lysosomes results in drug payload release and its intrinsic cytotoxicity. In addition to cytotoxicity, ADC drug payloads can sensitize tumor cells to ionizing radiation induced damage and kill.

Based on the experience with cetuximab and radiotherapy, one could hypothesize that there is an advantage in combining potent cytotoxins with radiotherapy over signaling pathway inhibitors. Specifically, cytotoxic radiosensitizers provide two distinct opportunities to kill cancer cells: 1) additive intrinsic tumoricidal activity due to different mechanisms of tumor kill and 2) synergistic radiosensitization. The inherent single agent anti-tumor activity of cytotoxic chemotherapies should not be discounted when designing molecularly targeted radiosensitization strategies. However a fundamental problem in giving increasingly potent cytotoxins with IR remains tumor specific drug delivery to minimize peri-tumoral and systemic toxicities. Antibody drug conjugates (ADCs) with their receptor-directed drug delivery offers a solution for molecularly targeted cytotoxic radiosensitization (Fig. 2).

## Antibody drug conjugates

ADCs are emerging as effective targeted systemic therapies for select cancer patients^21,22^. ADCs are constructed with modular architecture, consisting of three components: 1) an antibody with membrane receptor affinity, 2) a drug payload, and 3) a chemical linker coupling drug with antibody (Fig. 2a, b). ADCs split the roles of targeting and killing into two distinct molecular tasks. ADC tumor-targeting is dictated by the antibody moiety recognizing specific membrane receptors overexpressed on cancer cells. Tumor kill is mediated by the attached drug payload. Upon binding to cell membrane target receptors, ADCs are internalized through receptor-mediated endocytosis followed by enzymatic cleavage or chemical degradation of the linker within lysosomes liberating drug from antibody (Fig. 2c). ADCs can deliver potent cytotoxins with spatial precision in a receptor restricted fashion while simultaneously minimizing normal tissue damage.

Clinically successful ADCs were initially limited to leukemias and lymphomas, but efficacy across diverse solid tumor histologies is being demonstrated, expanding the number of approved ADCs (Table 2). Membrane receptors validated as ADC targeting beacons include HER-2, nectin-4, trop-2, folate receptor α and tissue factor (TF) receptors^21^. Relevant to radiosensitization, these receptors are found on tumors treated with chemo-radiotherapy (Table 1)^23–29^. The potent cytotoxic drug payloads of ADCs fall into three classes: 1) anti-tubulins (auristatins, maytansinoids), 2) DNA binders (pyrrolobenzodiazeines) and 3) topoisomerase I inhibitors (SN-38, exatecans)^22^. One of the pioneering ADCs was brentuximab-vedotin (Adcetris) whose design paved the way for additional approved ADCs sharing identical drug-linker chemistry (Fig. 2b)^30^. To construct brentuximab-vedotin, the anti-tubulin drug monomethyl auristatin E (MMAE) was conjugated to CD30 targeting antibody brentuximab through a maleimidocaproyl-valine-citrulline-p-aminobenzyloxycarbonyl (MC-VC-PABC) linker^31^. Upon binding CD30 expressing lymphoma cells, brentuximab-vedotin is internalized and cathepsins cleave the linker at the valine-citrulline dipeptide. This triggers self-immolation of the PABC moiety resulting in release of free MMAE that arrests cells in G_2_/M.

**Table 2:** FDA approved ADCs for cancers treated with radiotherapy

ADC	Trade name	Approved indication	Receptor target	Linker	Drug payload	Drug action	DAR
Brentuximab vedotin	Adcetris	Hodgkin Lymphoma, anaplastic large cell lymphoma	CD30	Enzyme cleavable	MMAE	Microtubule inhibitor	4
Trastuzumab emtansine	Kadcyla	HER2+ breast cancer	HER2	Non-cleavable	DM1	Microtubule inhibitor	3.5
Polatuzumab vedotin	Polivy	DLBCL	CD79b	Enzyme cleavable	MMAE	Microtubule inhibitor	3-4
Enfortumab vedotin	Padcev	Urothelial cancer	Nectin-4	Enzyme cleavable	MMAE	Microtubule inhibitor	4
Trastuzumab deruxtecan	Enhertu	HER2+ breast, NSCLC, gastric, esophageal cancers	HER2	Enzyme cleavable	DXd	Topoisomerase I inhibitor	8
Sacituzumab govitecan	Trodelvy	Triple negative breast, urothelial cancers	Trop-2	Hydrolyzable	SN-38	Topoisomerase I inhibitor	7.6
Tisotumab vedotin	Tivdak	Cervical cancer	TF	Enzyme cleavable	MMAE	Microtubule inhibitor	4
Loncastuximab tesirine	Zylonta	DLBCL	CD19	Enzyme cleavable	PBD SG3199	DNA crosslinker	2.3
Disitamab vedotin	Aidixi	Gastric cancer	HER2	Enzyme cleavable	MMAE	Microtubule inhibitor	4

*ADC* Antibody drug conjugate, *DM1* Mertansine, *NSCLC* Non-small cell lung cancer, *DAR* Drug to antibody ratio, *DXd* Exatecan derivative, *PBD* Pyyrolobenzodiazepine, *DLBCL* Diffuse large B cell lymphoma, *MMAE* Monomethyl auristatin E, *TF* Tissue factor.

Theoretically, ADCs are the “magic bullets” that systemically locate and kill cancer cells while sparing normal tissues. However, the clinical experience with ADCs has revealed shortcomings, including toxicity and resistance^32–34^. ADC toxicities are classified as “on-target” or “off-target”. Expectedly, ADC targeted receptors are not exclusive to cancer cells. Therefore, ADCs bind non-cancerous cells in a receptor-specific “on-target” manner with the drug payload damaging normal tissues. In addition, “off-target” effects occur independent of target receptor engagement. Mechanisms for ADC “off-target” toxicity include: 1) linker instability prematurely releasing drug 2) non-specific ADC internalization by attaching non-targeted receptors, and 3) bystander effects occurring when ADC cleavage in target cells releases the drug from antibody constraint which then freely diffuses into adjacent non-cancerous cells. The cumulative clinical experience suggests “off-target” mechanisms drive toxicity since side effect profiles of different ADCs can be grouped together by drug payload and not targeting antibody^33^. In a recent meta-analysis, the incidence of significant ADC toxicities (grade ≥ 3) was reported to be 46.1% and included hematologic and neuropathy^35^.

**Fig. 3:**
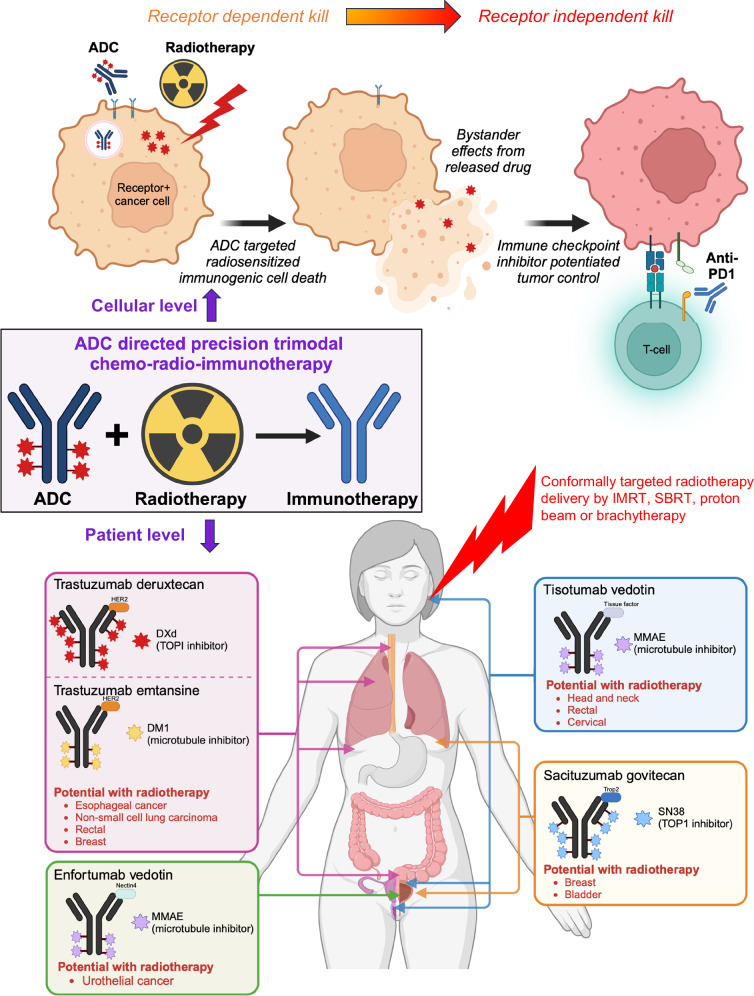
ADC directed trimodal chemo-radio-immunotherapy. Trimodal chemo-radio-immunotherapy treatment package consisting of systemically delivered radiosensitizing ADCs given together with conformal radiotherapy followed by immunotherapy. ADCs combined with radiotherapy produce receptor restricted radiosensitized immunogenic cell death depicted at the cellular level (top). After initial receptor mediated ADC internalization, linker cleavage releases free drug that can diffuse into surrounding tumor cells producing bystander cytotoxicity and radiosensitization in a receptor-independent manner. ADC drug payload radiosensitized tumor kill stimulates tumor immune infiltration and in concert with immune checkpoint inhibitors improves tumor control in a receptor-independent manner. Translation potential of ADC directed trimodal precision chemo-radio-immunotherapy with current clinically approved ADCs (bottom).

Resistance also curtails ADC clinical utility^36^. For example, tumor heterogeneity dictates target receptor expression varies within a tumor and adaptive receptor expression reduction selecting for low receptor expressing cells can render ADCs ineffective. Cells with high membrane receptor density are sensitive to ADC kill while those with lower receptor density are resistant. In the case of HER2 targeted ADCs, solutions to overcome receptor heterogeneity include use of tyrosine kinase inhibitors (e.g. neratinib, poziotinib) to modulate HER2 availability and improve ADC response by T-DM1^37,38^. Alternative to modulating receptor targets, drug payload can be optimized to maximize tumor kill. To optimize their therapeutic index, MMAE-ADCs are constructed with a drug antibody ratio (DAR) of 4 due to instability and hydrophobic aggregation (Table 2)^39^. Newer topoisomerase 1 inhibitor exatecan and SN-38 ADCs have a DAR of up to 8^21,40^. Having twice as much drug delivered per each ADC-receptor engagement can improve the efficacy against lower receptor density expressing tumors^41,42^. By choosing membrane permeable ADC payloads, therapeutic extension to neighboring receptor-negative cancer cells by bystander effect ADC release of drug from receptor-positive cells can overcome tumor receptor heterogeneity (Fig. 3)^43,44^. The concept of synthetic lethality has also been applied to ADCs by combining PARP inhibitors with topoisomerase I inhibitor ADCs^45^. Finally, dual payload ADCs with 2 different drug payloads attached to an antibody can overcome receptor heterogeneity and therapy resistance in pre-clinical cancer models^46^. Such a strategy mirrors the success of combinatorial chemotherapy with the precision of ADC targeting. Combining focal radiotherapy with systemically delivered ADCs offers an alternative approach to improve the therapeutic index of ADCs given their different mechanisms of action along with the potential to yield therapeutic synergies.

## ADC targeted radiosensitization

One of the most common ADC drug payloads is MMAE (Table 2). MMAE belongs to a family of auristatins that are synthetic analogues of naturally occurring dolastatin 10^47^. Auristatins block tubulin polymerization causing G_2_/M cell cycle arrest and cell death. Combined with radiotherapy, MMAE has the dual property of radiosensitizing cells in addition to its intrinsic cytotoxicity. In radiosensitization assays, MMAE as a free drug increased IR induced DNA damage signaling, DNA double stranded breaks and cell kill compared to irradiation alone^48,49^. Translationally, ADC tumor-targeted MMAE radiosensitization has been established with multiple ADCs, while differing in targeting antibody all sharing an identical MMAE payload^50–52^. Proof of concept studies were established with tool compounds where MMAE was conjugated to EGFR or HER2 targeting antibodies using the standard MC-VC-PABC linker of brentuximab-vedotin (Fig. 2b). While free MMAE indiscriminately radiosensitized independent of tumor receptor status, antibody conjugation specified MMAE radiosensitization to target-receptor enriched tumors, i.e. cetuximab conjugated MMAE radiosensitized EGFR expressing but not EGFR negative cancer cells while trastuzumab conjugated MMAE radiosensitized HER2 expressing but not HER2 negative cancer cells^50^. Expanding the radiosensitizing repertoire of pre-clinical MMAE based ADCs, HER3 is emerging as a clinically viable ADC target receptor^53,54^. MMAE conjugated to anti-HER3 antibody restricted radiosensitization to HER3 expressing human cancer cells and tumor xenografts^51^. Progressing to FDA approved ADCs with MMAE payload, pre-clinical studies have validated therapeutic synergy of MMAE based ADCs with IR. In head and neck cancer, tisotumab-vedotin added to chemo-radiotherapy improved tumor xenograft control^55^. In bladder cancer, enfortumab-vedotin combined with IR increased and tumor xenograft control^56^. Interestingly, clinical case reports of exceptional tumor responses in patients treated with radiotherapy combined with MMAE containing brentuximab-vedotin or enfortumab-vedotin have been reported^57,58^.

While initial IR and ADC research centered on MMAE, other ADC drug payloads are being evaluated. Corroborating auristatin based ADC radiosensitization with a different anti-tubulin class, enhanced irradiated tumor kill was demonstrated with T-DM1 (Kadcyla)^21,23^. T-DM1 consists of a maytansinoid (DM1) attached to trastuzumab. Similar to MMAE, free maytansinoid indiscriminately radiosensitized^50^. In contrast, T-DM1 showed selective cytotoxicity and targeted radiosensitization for HER2-expressing but not HER2-negative cancer cells. Extending beyond anti-tubulin based ADCs, interactive tumor xenograft kill has been shown with the topoisomerase I inhibitor ADC sacituzumab-govitecan in combination with IR^56^.

As with conventional chemo-radiotherapy and given the side effects of ADCs, co-delivering ADCs with radiotherapy raises concerns of increased toxicity. Such normal tissue damage adjacent to irradiated tumor volumes could cause non-overlapping damages of ADC and IR or from ADC radiosensitization. As discussed above, peri-tumoral normal tissues are susceptible to both receptor-dependent and receptor-independent toxicities of ADCs that may increase in combination with radiotherapy. In the case of bystander based toxicity, ADC internalization by receptor-expressing tumor cells releases drug that can then diffuse into adjacent normal tissues where subsequent tumor irradiation risks the potential for radiosensitized damage to peri-tumoral normal tissues. As a solution to mitigate this risk, the auristatin derivative monomethyl auristatin F (MMAF) can reduce normal tissue bystander radiosensitization. MMAF has a negative charge resulting in decreased cell permeability compared to MMAE as free drug^59^. Therefore, MMAF is reliant on antibody conjugation for intracellular uptake, cytotoxicity and radiosensitization and may have offer improved tumor selective radiosensitization^44,48^.

With the increased use of ADCs in cancer patients, the safety profile of ADCs with radiotherapy is being established. A meta-analysis of the safety of T-DM1 with concurrent radiotherapy in the local-regional treatment of non-metastatic breast cancer showed favorable toxicity profile in the irradiated region (i.e. acute skin effects and radiation-induced pneumonitis)^60^. In a case report of patients with locally advanced bladder cancer treated with enfortumab-vedotin and radiotherapy, no increased radiation related adverse effects were found^58^. In contrast, experience in the brain metastases setting suggests caution is warranted due to increased radionecrosis seen with T-DM1^60^. Alternatively, T-DXd with radiotherapy in metastatic breast cancer patients appears to have an acceptable toxicity profile^61^. In summary, therapeutic synergies of IR with anti-tubulin and topoisomerase I inhibitor based ADCs provides a scientific basis for clinically testing ADCs with radiotherapy to achieve spatially precise tumor radiosensitization that can improve the therapeutic index of radiotherapy compared to conventional cytotoxic chemotherapies (Fig. 2c). However, anatomic considerations of radiotherapy delivery and choice of ADC drug payload need to be accounted for to mitigate treatment toxicities, particularly with intracranial irradiation.

## Potentiating immunotherapy by ADC radiosensitization

Immunotherapies have revolutionized cancer treatment^62^. However, durable responses to immune checkpoint inhibitors are limited to patient subsets spurring combinatorial testing^63^. ADC-immunotherapy combinations are being evaluated given non-overlapping mechanisms of tumor kill that may overcome resistance to each individual modality while maintaining an acceptable safety profile^22^. Proof of concept has been demonstrated in combining enfortumab-vedotin (EV) with pembrolizumab that improved outcomes over standard chemotherapy in metastatic urothelial cancer patients (KEYNOTE-A39 trial)^64^. EV is a nectin-4 targeted ADC with MMAE attached through MC-VC-PABC linkage (Table 2)^25^. This trial has been hailed as a landmark since it provided the first evidence of ADC with immunotherapy outperforming chemotherapy. In addition, ADC induced immunogenic cell death can release tumor-specific neoantigens and/or shift immunosuppressive tumor-immune microenvironments towards immune-activated^65^. Disproportionate to the number of clinical trials, there are few mechanistic pre-clinical studies of how ADCs modulate the tumor immune microenvironment or interact with immunotherapies due to species specificity of clinically approved ADCs^48,66,67^. In one example, immune-competent mice treated with ADC U3-1402 (HER3 targeted-DXd) had increased tumor infiltration by both the innate and adaptive arms of immune system^66^. Moreover, U3-1402 combined with PD-1 inhibitor improved tumor control. As an example to overcome immune-resistance, ADC enapotamab vedotin (AXL targeted-MMAE), showed interactive tumor kill in combination with T-cells and anti-PD-1 treatment in immunotherapy-refractory murine cancer models^67^. From a safety standpoint, meta-analysis of trials evaluating ADCs with immune checkpoint inhibitors have shown toxicity rates comparable to ADC monotherapy with additive respiratory and gastrointestinal toxicities^68^.

Given IR’s unique advantage of spatial-temporal targeted dose deposition, radiotherapy has been proposed as a means to stimulate immune responses that provides a complementary strategy to widen radiotherapy’s therapeutic index (Fig. 1)^16,69–71^. Initial approaches focused on large 8-20 Gy doses to induce immunogenic cell death and generate abscopal effects, i.e. tumor control at metastatic sites out of the irradiated field^72–75^. However, focal ablative radiotherapy rarely generates systemic abscopal effects in patients^76–78^. Alternatively, immunotherapy with chemo-radiotherapy using conventionally fractionated lower IR doses is also being tested. More often adding immunotherapy to concurrent chemo-radiotherapy has failed to show benefit^78,79^. Of note, two trials highlight the role of immune checkpoint inhibitors with chemo-radiotherapy. In KEYNOTE-A18 trial, concurrent pembrolizumab (anti-PD-1) improved outcomes in cervical cancer patients treated with standard chemo-radiotherapy^80^. In the PACIFIC trial, adding adjuvant durvalumab (anti-PD-L1) after chemo-radiotherapy improved survival in non-small cell lung cancer patients^81^. With the caveats of different tumor types and timing of immune checkpoint inhibitor, these trials provide the first evidence of efficacy in combined immunotherapy with chemo-radiotherapy. While the immunotherapies and radiotherapy delivery are state of science, chemotherapies in these trials remain non-targeted. To determine if molecularly targeted radiosensitization can potentiate immunotherapies, inhibitors to the DNA damage response and repair pathways are being tested^17,82^. ATM and ATR are key DNA damage sensing kinases and modulate irradiated cell fate. In preclinical models, ATM and ATR inhibitors together with IR created a more favorable anti-tumor immune microenvironment (e.g. increased cytotoxic T-cells, decreased Tregs), increased T-cell receptor diversity, generated immunologic memory and combined with immunotherapies improved tumor control^83–86^.

In contrast to pathway specific non-cytotoxic DNA damage repair inhibitors, ADCs offer a molecularly targeted cytotoxic radio-immunosensitzation approach. Given the established track record of cytotoxic concurrent chemo-radiotherapy (Table 1) and the limited clinical success of chemo-radiotherapy with immunotherapies, opportunities exist to test if ADC-based cytotoxic radiosensitization can optimize immune system engagement by spatially restricting cytotoxic radiosensitizer delivery to tumor cells. As noted, there are few pre-clinical studies on the interplay of ADCs with tumor-immune microenvironment due to the species specificity of ADC targeting antibodies. Therefore, pre-clinical ADC research has overwhelmingly used human tumor xenografts grown in immune-deficient mice with the drawback that such models fail to define if ADCs sculpt the tumor-immune microenvironment or interact with immunotherapies. To overcome these hurdles and interrogate how ADCs impact the tumor-immune interplay, alternative drug delivery platforms can allow for investigating ADC drug payloads in immune-competent murine cancer models. Studies with MMAE and IR highlight such an approach. Complementary tumor-targeted MMAE peptide-drug conjugates and ADCs were synthesized by coupling the identical MC-VC-PABC-MMAE drug-linker moiety to activable cell penetrating peptide (ACPP) or anti-HER3 antibody (CDX3379), generating ACPP-MMAE and CDX3379-MMAE (Fig. 2b)^48^. For translational relevance, ACPP-MMAE and CDX3379-MMAE share identical drug-linker chemistry of clinical ADCs carrying MMAE (i.e. brentuximab-vedotin, enfortumab-vedotin and tisotumab-vedotin) and only vary in how MMAE is tumor-targeted^25,29,30^. HER3-targeted ADCs have an advantage in that anti-HER3 antibodies can recognize both human and murine HER3 allowing for simultaneous investigation in human xenograft and murine syngeneic tumors established in immune-deficient and immune-competent mice^48,66,87^. However, there are few murine syngeneic tumors expressing HER3. Alternate to ADCs, ACPP drug delivery technology provides a receptor-independent mechanism to deliver intravenously injected MMAE to tumors and interrogate interactive effects with radiotherapy and immunotherapies using well-characterized syngeneic murine tumor models^48,49^. Constructing complementary ACPP- and ADC-MMAE conjugates also provides opportunity to rigorously focus on if drug payload radiosensitization modulates the tumor-immune microenvironment^48^. Tumor-targeted MMAE (ADC or ACPP conjugate) combined with IR increased tumor immune infiltration of CD45 + , CD8 + T-cells, macrophages and neutrophils, compared to IR or MMAE alone. In the case of T-cells, a temporal increase in CD8 + T-cell tumor infiltration occurred 1 week after MMAE and radiotherapy suggesting an optimal time for adjuvant immunotherapy may exist. Mechanistically, the durable tumor control achieved by MMAE radiosensitization was governed by adaptive immune responses in a CD8 + T-cell dependent manner. Moreover, MMAE radiosensitization induced immunogenic cell death resulting in immunologic memory capable of rejecting tumor re-challenge. Translationally, MMAE tumor radiosensitization potentiated immune checkpoint inhibitor efficacy. Using sub-therapeutic doses of individual therapies, combining tumor targeted-MMAE, IR and anti-PD-1 therapy produced durable long term control in multiple tumor types. Using lower individual doses highlights an advantage of combining ADCs with IR and immunotherapies to reduce individual toxicities by leveraging therapeutic synergies. The ability of tumor-targeting peptide and antibody conjugates of MMAE to phenocopy each other with regards to radiosensitized tumor immune infiltration and potentiated immune checkpoint inhibitor tumor control suggests the findings are intrinsic to MMAE radiosensitization and not unique to specific MMAE-ADCs that may be broadly exportable to all vedotin-based ADCs (Table 2, Fig. 3).

## Conclusions

Concurrent chemo-radiotherapy resulted in a paradigm shifting approach to locally advanced cancers over forty years ago^1^. In the ensuing decades, radiotherapy and immunotherapy advances have progressed towards precision oncology^82^. In contrast, chemotherapies given concurrently with radiotherapy remain classical non-targeted cytotoxins. ADCs offer a solution for molecularly targeting dual action cytotoxic radiosensitizing drugs to be delivered with spatial precision to irradiated tumors. Cooperative tumor kill by ADCs and radiotherapy can stimulate tumor immune infiltration and potentiate immunotherapies. Such trimodal ADC-radio-immunotherapy combinations may widen the therapeutic index of ADCs, radiotherapy and immune checkpoint inhibitors to not only maximize tumor control in the irradiated field but also generate systemic anti-tumor immune responses and abscopal responses. Translationally, many cancers treated with chemo-radiotherapy have subsets of patient subsets expressing receptors targetable by FDA approved ADCs providing opportunities for clinically testing ADC directed chemo-radiotherapy against standard non-targeted cytotoxic chemotherapies (Table 1, Fig. 3).

We highlighted the ADC drug payload MMAE since it has dual advantages of being both a potent cytotoxin and a radiosensitizer^49^. Since MMAE radiosensitized tumor control stimulated immune infiltration and potentiated immune checkpoint inhibitors irrespective of drug delivery platform (i.e. antibody or cell penetrating peptide), it lends credence to the potential for therapeutic extension across MMAE containing ADCs including the three currently approved (i.e. brentuximab-vedotin (Adcetris), enfortumab-vedotin (Padcev) and tisotumab-vedotin (Tivdak)) in combination with radiotherapy and immune checkpoint inhibitors^25,29,30,48^. As noted, case reports of exceptional tumor responses have been reported in patients treated with radiotherapy in combination with brentuximab-vedotin or enfortumab-vedotin^57,58^. Such provocative observations provide an impetus to translationally evaluate ADCs within chemo-radio-immunotherapy regimens in the upfront potentially curable locally advanced cancer setting. And while the pre-clinical data in combination with IR has centered on anti-tubulins (MMAE or DM-1), ADCs with topoisomerase I inhibitor payloads (DXd and SN-38) are gaining traction and showing efficacy in diverse solid tumors using a variety of targeting antibodies (Table 2). Evaluating topoisomerase I inhibitor ADCs in combination with radiotherapy and immunotherapies may reveal similar and/or unique therapeutic synergies compared to anti-tubulin based ADC radio-immunosensitization. Moving beyond focal external beam radiotherapy, pairing ADCs with radiopharmaceuticals could achieve systemic radiosensitization in the metastatic setting. For example in metastatic prostate cancer patients, beta-emitting lutetium-177-PSMA-617 (Pluvicto) has recently shown modest efficacy, but dose escalation will be limited by systemic radioisotope toxicity and resistance (in part receptor based)^88^. Co-delivering a PSMA targeted ADC with lutetium-177-PSMA-617 could radiosensitize metastatic cancer cells systemically at all sites of disease and not be limited by focal external beam radiotherapy delivery.

Rationally integrating ADCs with radiotherapy and immunotherapies can leverage their complementary strengths and overcome the limitations of ADC efficacy imposed by tumor heterogeneity and resistance. Trimodal ADC-radio-immunotherapy may result in therapeutic extension beyond receptor-targeted kill through bystander radiosensitization and invigorating anti-tumor immune responses independent of ADC target receptor (Fig. 3). It could be envisioned that tumor regions expressing elevated levels of ADC target receptor will readily internalize ADCs resulting in receptor-dependent tumor cell kill and radiosensitization. After eliminating this receptor-rich tumor fraction, the bystander effect is elicited with released free drug taken up by neighboring tumor cells with lower ADC target receptor expression. Since radiotherapy is routinely delivered daily over consecutive weeks for locally advanced cancers, the bystander effect could be spatially amplified by continued free drug radiosensitization of neighboring tumor cells. Finally, ADC drug payload radiosensitized tumor kill (both ADC receptor-dependent and bystander receptor-independent) can induce immunogenic cell death, stimulating anti-tumor immune responses to tumor antigens independent of ADC target receptor to promote durable tumor control by optimally potentiating immunotherapies. In summary, the emerging pre-clinical and clinical work with ADCs provide a rationale for integrating them into the historically successful chemo-radiotherapy paradigm to progress from non-targeted cytotoxic chemotherapies toward spatially precise ADC directed trimodal chemo-radio-immunotherapy combinations.

## Data Availability

No datasets were generated or analysed during the current study.
